# Restoration of DAP Kinase Tumor Suppressor Function: A Therapeutic Strategy to Selectively Induce Apoptosis in Cancer Cells Using Immunokinase Fusion Proteins

**DOI:** 10.3390/biomedicines5040059

**Published:** 2017-10-04

**Authors:** Mehmet Kemal Tur, Adebukola K. Daramola, Stefan Gattenlöhner, Marco Herling, Shivan Chetty, Stefan Barth

**Affiliations:** 1 Institute of Pathology, University Hospital, Justus Liebig University Giessen, 35390 Giessen, Germany; Mehmet.K.Tur@patho.med.uni-giessen.de (M.K.T.); stefan.gattenloehner@patho.med.uni-giessen.de (S.G.); 2 South African Research Chair in Cancer Biotechnology, Institute of Infectious Disease and Molecular Medicine, Department of Integrative Biomedical Sciences, Faculty of Health Sciences, University of Cape Town, Cape Town 7700, South Africa; dara.adebukola@gmail.com (A.K.D.); CHTSHI005@myuct.ac.za (S.C.); 3 Laboratory of Lymphocyte Signaling and Oncoproteome, Excellence Cluster for Cellular Stress Response and Aging-Associated Diseases, University of Cologne, 50923 Köln, Germany; marco.herling@uk-koeln.de; 4 Department I of Internal Medicine, Center for Integrated Oncology Köln-Bonn, and CECAD, University of Cologne, 50923 Köln, Germany

**Keywords:** cancer immunotherapy, death-associated protein kinases (DAPk), apoptosis inducers, humanised cytolytic fusion proteins (hCFPs)

## Abstract

Targeted cancer immunotherapy is designed to selectively eliminate tumor cells without harming the surrounding healthy tissues. The death-associated protein kinases (DAPk) are a family of proapoptotic proteins that play a vital role in the regulation of cellular process and have been identified as positive mediators of apoptosis via extrinsic and intrinsic death-regulating signaling pathways. Tumor suppressor activities have been shown for DAPk1 and DAPk2 and they are downregulated in e.g., Hodgkin’s (HL) and B cell lymphoma (CLL), respectively. Here, we review a targeted therapeutic approach which involves reconstitution of DAPks by the generation of immunokinase fusion proteins. These recombinant proteins consist of a disease-specific ligand fused to a modified version of DAPk1 or DAPk2. HL was targeted via CD30 and B-CLL via CD22 cell surface antigens.

## 1. Introduction

Apoptosis is an important cellular event that allows multi-cellular species to get rid of unwanted, damaged, infected, or cancerous cells [1]. It is a vital cellular mechanism necessary for the maintenance of tissue and body homeostasis. Notably, cells undergo apoptosis through two major pathways: the extrinsic or death receptor pathway and the intrinsic or mitochondria pathway [2]. Under normal conditions, as cells undergo tumorigenesis they are subjected to various stress conditions such as unbalanced hyper-proliferative signals, which trigger various mechanism of programmed cell death. These conditions include constant selective pressure, such as lack of oxygen [3], depletion of growth/survival factors, and attack from the immune system and death, mostly by anoikis due to loss of cell-matrix interactions [4]. Unfortunately, death is often not the case, as cancer cells can manipulate the regulatory mechanisms put in place to control apoptosis for their own survival and progression. In most cases, cancer cells benefit from hyperregulatory of DNA to silence pro-apoptotic genes or initiate epigenetic modifications that activate anti-apoptotic genes [5]. One common mechanism utilized by malignant cells to selectively silence genes is the methylation of the CpG islands in the promoter regions of genes via the addition of a methyl group to the cytosine ring to form methylcytosine [6]. Some examples of tumor suppressor genes reportedly affected by DNA methylation include p16^INK4a^, p15^INK4b^, p14^ARF^, Von Hippel-Lindau (VHL), APC, and E-cadherin [7,8,9]. Also in this category are the death associated protein kinase (DAPk) genes, which play an essential role in the apoptosis signaling pathway during tumorigenesis [10]. The promoter region of DAPk1 and DAPk2 (members of the DAPk gene family) are reportedly hypermethylated in multiple tumor types [11,12] with the highest frequency of DAPk methylation detected in B-cell lymphoma [4]. Based on this evidence, DAPk fusion proteins were developed and subsequently shown to restore lost tumor suppressor functions in DAPk downregulated cancer cells. We describe in this review preclinical outcomes of studies reconstituting active DAPk in the fashion of an immunokinase fusion proteins for targeted cancer therapy. 

## 2. Death Associated Protein Kinases (DAPk)

The death-associated protein kinases (DAPk) are a family of five Ser/Thr kinases whose members (DAPk1, DAPk2, ZIPk, DRAK-1, and DRAK-2) constitute one of the largest and the most functionally diverse gene families [13]. They act by adding phosphate groups to substrate proteins to modify serine, threonine, tyrosine, histidine, arginine, lysine, glutamic acid, or aspartic acid residues. By this, they direct the activity, localization, and overall function of many proteins, regulating the activity of almost all cellular processes [13]. They are required for the induction of cell death by multiple death signals, including those generated by death receptors, cytokines, matrix detachment, and hyper-proliferation [14,15,16]. The loss of DAPk1 and DAPk2 expression in multiple types of human cancer ignited the profound interest in the kinase family regarding their function and involvement in various diseases [17]. 

## 3. Structure and Function

The DAPk family of proteins are known to share some level of homology in their protein structure. Three closely related homologues include DAPk1, DAPk2, and ZIPk, which are sub-grouped into a common kinase sub-family due to the high degree of conservation within their catalytic domains. In humans, the catalytic domain of DAPk2 and ZIPk share 83% and 80% similarity at the amino acid level with DAPk1 [14]. The remaining members of the group—human DRAk-1 and DRAk-2 (DAPk-related apoptosis-inducing protein kinase-1 and -2)—are only 50% identical to the kinase domain of DAPk1 [14]. This section briefly describes the structure and functional properties of DAPk1, DAPk2 and ZIPk.

DAPk1 was first discovered in the 1990s as a functional mediator of interferon (IFN)-induced cell death in HeLa cells [18]. It is the largest of all members of the DAPk family with a molecular weight of 160 kDa [19]. This relatively large size of DAPk1 in comparison to DAPk2 and ZIPk of 42 and 55 kDa respectively is associated to its unique and large C-terminal extension. The catalytic domain of all three proteins (DAPk1, DAPk2, and ZIPk) lie at the N-terminus and are made up of 11 sub-domains [20]. At the upper lobe of the catalytic domain is a positively charged loop enriched with 12 amino acids (aa 45–56) [21], which has been shown to mediate DAPk1 homodimerization [22]. In addition, this loop does mediate the interaction between DAPk1 and ZIPk, an interaction that leads to phosphorylation and activation of ZIPk by DAPk1 [23]. Though of high proximity to the catalytic domain, this loop is not directly involved in substrate binding as mutation of its basic residues did not affect the *K*_m_ of a peptide substrate [24].

**Figure 1:**
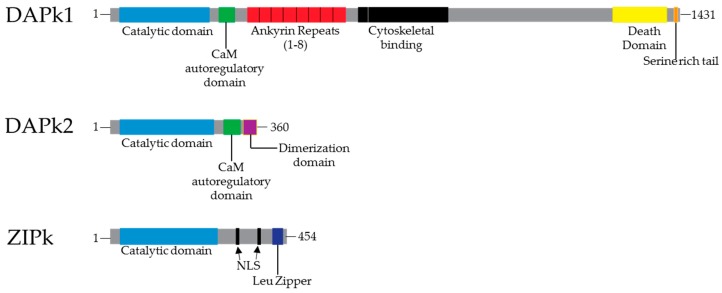
Schematic representation of the multi-domain organization of death associated protein kinase DAPk1 (death-associated protein kinases 1), and ZIPk. The catalytic domain, a death domain, and ankyrin repeats, which may mediate its interaction with other proteins. The cytoskeleton-binding region is responsible for DAPk1 intracellular localization to actin microfilaments. DAPk1 and DAPk2 are activated by a rise in cytosolic calcium concentrations resulting from cellular stresses, through binding of calcium-activated calmodulin [33]. NLS = nuclear localization signal.

At the C-terminus of the catalytic domain of DAPk1 and DAPk2 is a Ca^2+^/calmodulin (CaM) autoregulatory domain (Figure 1) that inhibits catalytic activity by binding to the catalytic cleft and by so doing prevents substrate binding [19]. Hence, the activation of DAPk1 and DAPk2 requires the presence and binding of Ca^2+^-activated CaM to the autoregulatory domain. This event pulls the autoregulatory domain away from the catalytic cleft, enabling substrate phosphorylation. Notably, activation of DAPk1 and DAPk2 is further enhanced by dephosphorylation of Ser308, which increases the affinity of DAPk1 and DAPk2 for CaM [25,26]. On the other hand, the regulation of ZIPk differs from that of DAPk1 and DAPk2 as it lacks the Ca^2+^/CaM autoregulatory domain. Instead, it possess an inhibitory C-terminal amino acid sequence that contains a nuclear localization signal (NLS) and a leucine zipper [27].

The downstream of the DAPk1 Ca^2+^/CaM autoregulatory domain includes other extracatalytic domains. These includes eight ankyrin repeats which are necessary for proper localization of DAPk1 to the actin filaments. The ankyrin repeats are also described to facilitate degradation of DAPk1 via the ubiquitin-proteasome pathway [20]. Located between residues 695–702 is the ROC-COR (Ras of complex proteins, C-terminal of ROC) domains, also called the cytoskeletal localization domain [27]. The ROC-COR domains are known to promote DAPk1 guanosine triphosphate (GTP) binding in an event believed to regulate DAPk1 activity. The ROC-COR domains also mediate protein–protein interactions (for example, with the phospho-Ser/Thr-directed peptidyl prolyl isomerase 1 (Pin1)). By phosphorylating Pin1, DAPk1 inactivates Pin1’s cellular functions, including its ability to induce cellular transformation [19].

The C-terminus of DAPk1 also contains a death domain common to many apoptosis-promoting proteins and located between residues 1312–1396. This death domain does mediate several functions including: (1) the interaction between DAPk1 Ser735 and the extracellular signal-regulated kinase (ERK) within the cytoskeletal binding region, causing an increase in DAPk1 catalytic activity [28]; (2) mediate interaction with the death domain-containing protein UNC5H2 [29], which induces cell death when unbound to its ligand, netrin-1; and (3) mediate interactions that influence DAPk1’s stability by binding to proteins that promotes degradation of DAPk1 (e.g., KLHL20) [30]. In contrast, DAPk2 and ZIPk lack the ankyrin repeats, cytoskeletal binding, and the death domains seen in DAPk1 [31]. DAPk2 is expressed as a soluble cytoplasmic protein and contains 40 amino acid sequence C-terminal to its CaM autoregulatory domain. At the C-terminus is a dimerization domain that allows for its homodimerization. The structural features of ZIPk allows for its cytoplasmic and nuclear distributions, mainly by interacting with proteins that directly interact with actin filaments (e.g., Par-4) [32].

## 4. DAPk1 and DAPk2: Regulation and Signaling Pathway

It is expected that an understanding of the underlying molecular mechanisms regulating members of the DAPk family or triggering their associated death responses is essential to the development of DAPk based therapies to combat cancer and associated diseases [34]. As described earlier, DAPk1 and DAPk2 can be activated by several mechanisms, including binding of Ca^2+^-activated CaM, which allows the release of the catalytic domain from inhibition by the CaM autoregulatory domain, phosphorylation of ERK, and dephosphorylation of Ser308 [27]. Notably, deletion of the CaM-binding domain (∆CaM) from either DAPk1 or DAPk2, or substitution of Ser308 to Ala, has allowed the generation of constitutively active kinase mutant with stronger cell death effect than the wild type kinase [19,20,35]. In other instances, transcriptional and translational mechanisms have been described as means by which DAPk1 and DAPk2 can be activated. The presence of transcription factors (e.g., transforming growth factor-β (TGFβ)) and p53 activating agents (e.g., DNA damaging agents and oncogene expression) are known to enhance the expression of DAPk1 [36,37]. The work of Britschgi et al. has also shown that the transcription factor SP1 is indispensable for basic DAPk2 promoter activity and that E2F1 and KLF6 transcription factors are necessary for further DAPk2 regulation [38].

**Figure 2:**
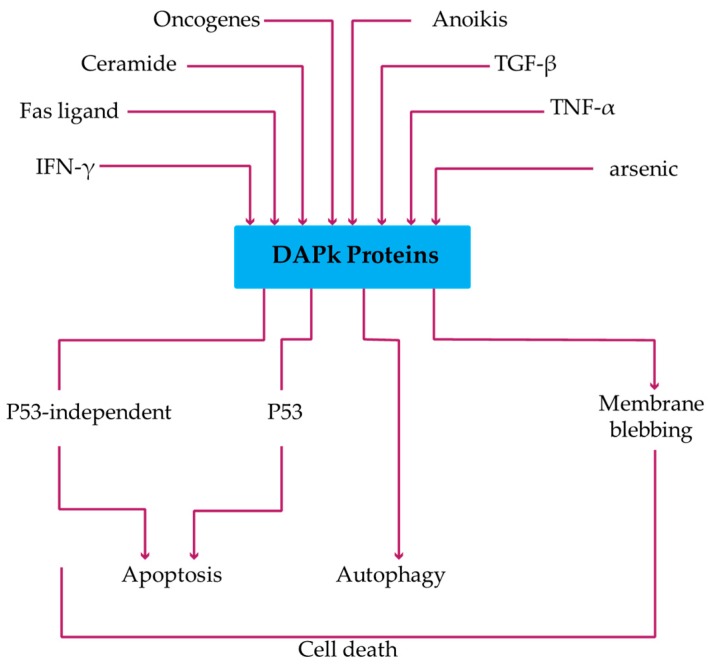
Activation of different DAPk signaling cascade by different stimuli. Based on the signal input and cell context, the DAPk family of genes play a crucial role is deciding the outcome of whether a cell survives or undergoes apoptosis. Activated DAPk proteins may initiate p53-dependent or independent apoptosis or mediate an autophagic programmed cell death [39].

Once activated, DAPk1 and DAPk2 can elicit a variety of death pathways depending on the cellular context. Accumulating data indicate that various stimuli may activate DAPk1 and DAPk2, including TNF-α, Fas ligand, ceramide, oncogenes, TGF-β, arsenic trioxide, and detachment from extracellular matrix (Figure 2) [39].

To induce apoptotic cell death, DAPk1 may require the activation of the p53 apoptotic pathway. In this case, induction of p19ARF by DAPk1 would allow inactivation of Mdm2 (a negative regulator of p53) to stabilize and activate p53. DAPk1 is a transcriptional target of p53 and the activation of DAPk1 eventually promotes its function by a positive feedback regulation between DAPk1 and p53 [40]. DAPk1 can also inactivate integrin to allow inactivation of FAK and the upregulation of p53. This inactivation of integrin also inhibit cell–matrix adhesion and ultimately promotes anoikis-type apoptosis [41]. Besides p53, DAPk1-induced apoptosis can be mediated by ERK1 and ERK2 [42]. ERK binds and phosphorylates DAPk1 to increase its catalytic activity but its retention in the cytoplasm by DAPk1 blocks its cell survival signal, thereby promoting apoptosis [28].

In other cellular settings, DAPk1 and DAPk2 have been linked to the activation of an alternate type of programmed cell death, referred to as Type II or autophagic cell death [43]. One such mechanism involves the phosphorylation of Beclin-1, which is required for autophagosome nucleation formation [44], and an interaction with the LC3 binding protein, MAP1B, which may regulate vesicle trafficking. A detailed molecular mechanisms of DAPk in regulating autophagy are reviewed here [45]. Though, less is known about the molecular events downstream of DAPk2 activation, phosphorylation of the regulatory light chain of myosin II (MLC) by activated DAPk2 has been reported to induce membrane blebbing and autophagy [46]. It is also believed that DAPk2 may function upstream of DAPk1 as the overexpression of a dominant negative DAPk1 reduced DAPk2 induced cell death [39]. Notably, the downstream pathways (Caspase- and p53-dependent vs. independent apoptosis) and biologic outcomes (i.e., apoptosis vs. autophagic cell death vs. necroptosis) mediated by DAPk1 and DAPk2 are dictated in a cell specific context and nature of the upstream signal [47]. For example, a caspase dependent cell death was induced in primary fibroblast after the overexpression of DAPk1 and DAPk2. While in HeLa, MCF-7, or HEK 293 cells, overexpression of DAPk proteins led to a caspase-independent cell death, with increased characteristics feature of autophagy (autophagic vesicles and autolysosomes) seen in HEK 293 or MCF-7 cells [48].

## 5. Loss and Restoration of DAPk Tumor Suppressor Function in Cancer

**Figure 3:**
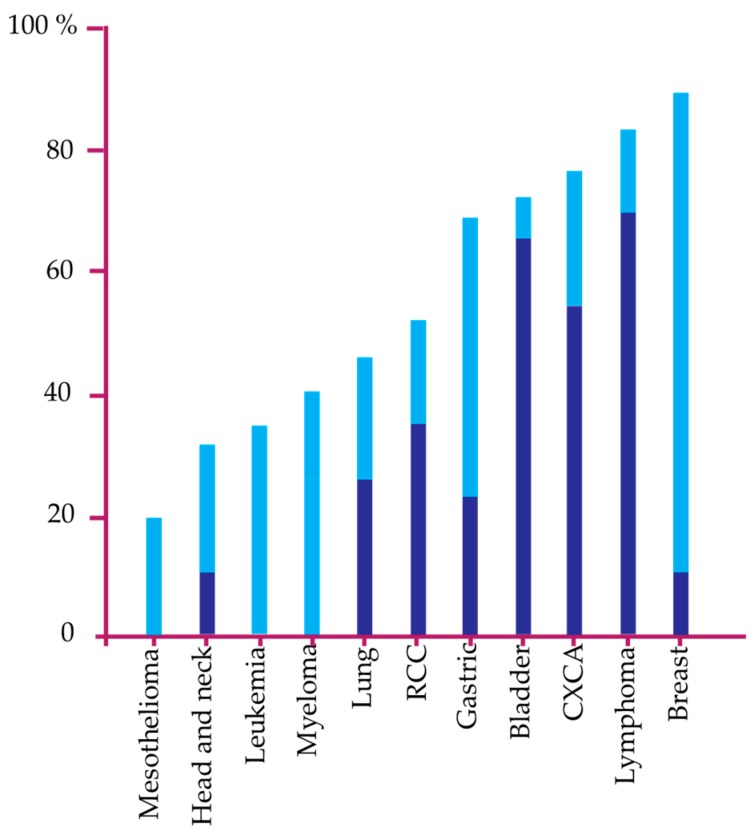
Percentages of DAPk gene methylation in different tumors. Minimal and maximal percentage of DAPk methylation is represented in dark blue and light blue, respectively, as identified by different studies [64,65,66,67,68,69,70,71,72,73,74]. RCC: renal cell cancer, CXCA: cervical cancer.

The inactivation of DAPk in cancer is usually a result of hypermethylation at the promoter of the DAPk gene rather than mutation [49]. The 5′-UTR of DAPk1 gene contains a CpG island in which hypermethylation leads to gene silencing and loss of expression [50,51]. The hypermethylation of DAPk1 and DAPk2 genes has been detected in several human tumors, albeit at varying methylation rates (Figure 3) [39]. Besides hypermethylation, microRNA (miRNA) which are noncoding RNA molecules can also downregulate DAPk levels by a miRNA-dependent mechanism. Chen et al., in a recent publication demonstrated that two miRNA (miR-103 and miR-107) can inhibit DAPk1 translation by targeting its 3′-UTR [52]. This was confirmed by a correlation between the increased expression of miR-103/107 and downregulation of DAPk1 in colorectal cancer (CRC) cell lines and CRC patients [53]. Additionally, downregulation of the transcriptional mediator complex subunit 1 (Med1) which interacts with transcriptional factor C/EBP-b to participate in the transcriptional activation of DAPk has been found to correlate with DAPk1 downregulation [42]. Futhermore, hypermethylation and transcriptional regulation, DAPk1 expression loss can also result from heterozygosity and homozygous deletion of DAPk1 as found in certain types of cancers [54,55]. In addition, the expression of DAPk1 in some tumors have shown that posttranslational mechanism may also inactivate DAPk1 [6]. For example, the tumor suppressor functions of DAPk1 can be inactivated by phosphorylation of DAPk1 by tyrosine kinase Src at Tyr491 and Tyr492 [56].

As methylation is a reversible process, agents capable of demethylating DNA (e.g., 5-azacytidine and 5-aza-2-deoxycytidine (Decitabine)) have been developed and tested. For example, demethylation of the DAPk1 gene by treatment with Decitabine was shown to restore DAPk expression and cell’s apoptotic sensitivity to IFN-γ [14]. This ability of Decitabine to also reactivate DAPk2 expression in Hodgkin lymphoma cell lines has also been demonstrated [57]. Unfortunately, despite their effectiveness, most demethylating drugs are chemically unstable and cannot be targeted to specific genes thus limiting their clinical use. Also, re-activation of protein expression by the treatment of epigenetically silenced genes with demethylating agents does not always result in increased amount of protein and has added to their drawbacks [58]. However, other approaches attempting to restore lost DAPk function have also been employed. This involves the transfection of cells with plasmids or viral vectors carrying the DAPk1 and DAPk2 cDNA [48,59,60]. While these approaches have allowed for the direct restoration of tumor suppressor activities [61,62], they are currently not optimized for specific tumor targeting in vivo. In recent years, a better understanding of the cellular/molecular activities of the DAPk family members has allowed the design of recombinant immunokinase fusion proteins that can restore apoptosis when targeted into the cytoplasm of cancer cells. This has led to the development of DAPk1 and DAPk2 based fusion proteins for the treatment of cancer [47,63].

## 6. Immunokinase-Based Fusion Proteins

**Figure 4:**
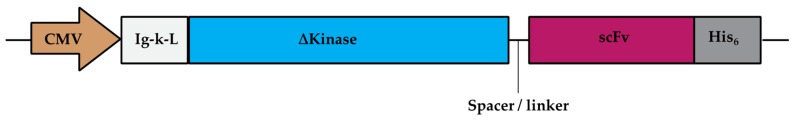
Schematic representation of an expression cassette for a recombinant immunokinase fusion protein. Under the expression of a strong CMV promoter, the N-terminal Igkappa leader sequence allows direction of expressed protein into the media of the expressing cell line. A C-terminal His_6_ tag sequence allows for affinity purification of the recombinant protein via an IMAC purification system.

Immunokinase fusion proteins are entirely made up of a targeting moeity specific for an unregulated surface marker on cancer cells and an effector domain capable of inducing a therapeutic benefit in tumor cells lacking kinase activity. In most cases, the targeting moeity is a single chain antibody fragment (scFv) genetically fused to a constitutively active mutant of a tumor suppressor kinase (Figure 4) [75]. Unlike the demethylating agents described above, immunokinase based fusion protein combine systemic targeted delivery and targeted activity to prevent off-target effects in normal cells expressing target antigens at physiological levels [75]. They are also entirely made up of human component, hence are not limited by immunogenicity drawbacks that affect the bacteria based fusion proteins. Other advantages of immunokinase-based fusion proteins include the ability to switch the targeting component to any ligand of choice and the ease with which they are expressed and purified from mammalian cell expression systems.

## 7. Restoring Apoptosis by Targeted Delivery of DAPk into Tumor Cells

### 7.1. DAPk1-Based Fusion Proteins for Chronic Lymphocytic Leukaemia

Mutants of DAPk1 lacking the CaM domain have allowed the generation of constitutively active DAPk1 [25] for immunotherapy. Using this approach, several advantages have come forward from the work of Lilienthal et al by targeting the CD22 receptor on chronic lymphocytic leukaemia (CLL) with the recombinant antibody fragment SGIII fused to a DAPk1 mutant lacking the native auto-inhibitory CaM domain [47]. Indeed, these findings clearly demonstrate that the immunokinase fusion protein (hCFP) termed DK1KD-SGIII specifically and efficiently killed CD22-positive cells of B-cell lymphoma lines (2 Burkitt’s, 1 FL/DLBCL, 2 CLL-derived) and primary CLL samples (IC_50_ ranging from 275 to 875 nM), without affecting CD22-negative cells from healthy donors or CLL-patients. Importantly, primary CD22 positive cells rapidly internalized DK1KD-SGIII within 45 min and a 48 h incubation of the hCFP with CD22 positive cell lines resulted in cell death and reduced viability in a dose dependent fashion. In contrast, the classically used CCL chemotherapeutic drug, fludarabine induced apoptosis irrespective of CD22-status showing the advantage of DK1KD-SGIII for future treatments. Importantly, it is also significant to note that DKIKD-SGIII efficiently killed CLL cells from patients resistant to clinical fludarabine treatment eliminating current limitation of conventional treatments. The mode of death was predominantly PARP-mediated and caspase dependent apoptosis. The identification of cleaved caspase 3 products and the expression of autophagy maker LC3B in cells treated with DK1KD-SGIII clearly highlight the therapeutic potential and functional similarities of recombinantly modified DAPK1 and wild-type.

### 7.2. DAPk2-Based Fusion Proteins for Hodgkin’s Lymphoma

Studies exploring the selective reconstitution of DAPk2 catalytic activity were first to demonstrate the proof-of-concept strategy of restoring tumor suppressive kinase activity by targeted delivery of actively constituted DAPk in the context of an immunokinase fusion protein. Expression of an immunokinase consisting of the transmembrane glycoprotein receptor of CD30 (CD30L) fused with a CaM negative DAPk2 mutant demonstrated apoptosis inducing capabilities in CD30 positive and DAPk2-negative tumor cell lines (L540 and L1236). The hCFP DAPk2-CD30L mediated in vitro cytotoxic activity with IC_50_ values of ~20 and ~63 nM in L540 and L1236 cells, respectively [63]. Its application in an in vivo Hodgkin’s lymphoma model also confirmed the therapeutic benefit of DAPK2-CD30L in SCID mice inoculated with L540 Hodgkin’s lymphoma cells. Treatment of mice with the maximum tolerable dose of 70 μg resulted in the following positive pre-clinical outcomes: (1) a highly significant overall survival time of greater than 175 days when compared with each control group (administered PBS and non-specific DAPk2-H22(scFv)) with 55 to 60 days mean survival times and (2) the presence of disseminated tumors were evident in the liver, lungs, spleen, and brain of mice in the control group after histological evaluation of prepared tissue sections [63]. Immunohistochemical analysis of tissue sections from PBS treated mice also confirmed the presence of the CD30 antigen, which in contrast was not detected in DAPk2-CD30L treated mice. This further supports of the recombinant immunokinase fusion protein to prevent tumor development in this mouse model.

## 8. Conclusions

The death-associated protein kinases 1 & 2 are representing pro-apoptotic proteins with tumor suppressor activities, share a highly-conserved N-terminal kinase domain, and a calcium/calmodulin-regulatory domain. Deletion of CaM is resulting in a constitutively active kinase with enhanced stimulation of programmed cell death. Several studies continue to report the downregulated levels of DAPk gene expression in various carcinomas therapeutic interventions or approaches that reinstate DAPK activity have the opportunity of introducing a new therapeutic principle (23–25). The first proof of concept studies have shown that a targeted delivery of a constitutively active DAPK2 to CD30-positive Hodgkin’s lymphoma cells selectively kills CD30-positive and DAPK2-negative but not CD30-positive and DAPK2-positive HL cells. This observation is of major importance for most tumor-associated targets as these are upregulated on tumor cells but also present on normal cells, potentially resulting in off-target effects of TAA specific therapeutics also hitting these normal cells. If these normal cells show normal expression of a tumor suppressor, they would not be hit by this therapeutic approach. Importantly, the outcomes of reconstituting DAPk in target cells, for example by the recombinant immunokinase DK1KD-SGIII (a revised DAPk1), is marked by a robust catalytic activity (in inducing apoptosis) in vitro, in vivo, and in primary cells from cancer patients [47]. However, it is of note that more data-driven studies across different DAPk deficient tumor types (head and neck cancers and other solid tumors) are needed to further confirm and strengthen the clinical potential of this emerging class of treatment. Unfortunately, low levels of DAPk2 promoter hypermethylation has also been found in normal peripheral blood cells. Particularly, the IgM-sub-populations of normal B-cells in healthy individuals are predominantly more DAPk hypermethylated when compared to other B-cells sub-populations [76]. The DK1KD-SGIII fusion construct described above was reported to also bind CD22(+) B-cells of healthy donors though further experiments are still required to evaluate DK1KD-SGIII enzymatic activity for death-induction of normal B-cells. It is also important to note that, unlike other novel cytotoxic enzymes of human origin (e.g., granzyme B and angiogenin) that are mostly limited as a result of upregulation of their endogenous inhibitors by target cells, DAPk1 and DAPk2 with its autoregulatory CaM domain removed theoretically are able to bypass tumor drug escape or resistance mechanisms. However, long-term in vivo studies could aid in gauging the potential ability of tumor cells to develop other escape mechanisms, as DAPk2 may also be regulated by oligomerization through their C-terminal tail (Tail) sequences [39].
